# Supplementary material for: A fault-intrusion-tolerant system and deadline-aware algorithm for scheduling scientific workflow in the cloud
Source: PeerJ Comput Sci. 2021 Nov 2;7:e747. doi: 10.7717/peerj-cs.747 (PMC8576554; doi:10.7717/peerj-cs.747)
Supplement: Supplemental Information 6 [file peerj-cs-07-747-s006.zip › Myproject1.1/docs/overview-tree.html]

Class Hierarchy


JavaScript is disabled on your browser.


- Overview
- Package
- Class
- Use
- Tree
- Deprecated
- Index
- Help

- Prev
- Next

- Frames
- No Frames

- All Classes

# Hierarchy For All Packages

Package Hierarchies:

- org.cloudbus.cloudsim,
- org.cloudbus.cloudsim.core,
- org.cloudbus.cloudsim.core.predicates,
- org.cloudbus.cloudsim.distributions,
- org.cloudbus.cloudsim.examples,
- org.cloudbus.cloudsim.examples.network,
- org.cloudbus.cloudsim.examples.network.datacenter,
- org.cloudbus.cloudsim.examples.power,
- org.cloudbus.cloudsim.examples.power.planetlab,
- org.cloudbus.cloudsim.examples.power.random,
- org.cloudbus.cloudsim.lists,
- org.cloudbus.cloudsim.network,
- org.cloudbus.cloudsim.network.datacenter,
- org.cloudbus.cloudsim.power,
- org.cloudbus.cloudsim.power.lists,
- org.cloudbus.cloudsim.power.models,
- org.cloudbus.cloudsim.provisioners,
- org.cloudbus.cloudsim.util,
- org.workflowsim,
- org.workflowsim.clustering,
- org.workflowsim.clustering.balancing,
- org.workflowsim.clustering.balancing.methods,
- org.workflowsim.clustering.balancing.metrics,
- org.workflowsim.examples,
- org.workflowsim.failure,
- org.workflowsim.planning,
- org.workflowsim.reclustering,
- org.workflowsim.scheduler,
- org.workflowsim.utils

## Class Hierarchy

- java.lang.Object
  - org.workflowsim.clustering.AbstractArrayList
  - org.cloudbus.cloudsim.network.datacenter.AppCloudlet
    - org.cloudbus.cloudsim.examples.network.datacenter.TestBagofTaskApp
    - org.cloudbus.cloudsim.network.datacenter.WorkflowApp
  - org.workflowsim.utils.ArgumentParser
  - org.workflowsim.clustering.balancing.methods.BalancingMethod
    - org.workflowsim.clustering.balancing.methods.ChildAwareHorizontalClustering
    - org.workflowsim.clustering.balancing.methods.HorizontalImpactBalancing
      - org.workflowsim.clustering.balancing.methods.HorizontalDistanceBalancing
    - org.workflowsim.clustering.balancing.methods.HorizontalRandomClustering
    - org.workflowsim.clustering.balancing.methods.HorizontalRuntimeBalancing
    - org.workflowsim.clustering.balancing.methods.VerticalBalancing
  - org.workflowsim.planning.BasePlanner (implements org.workflowsim.planning.PlannerInterface)
    - org.workflowsim.planning.RandomPlanner
  - org.workflowsim.scheduler.BaseScheduler (implements org.workflowsim.scheduler.SchedulerInterface)
    - org.workflowsim.scheduler.DataAwareScheduler
    - org.workflowsim.scheduler.FCFSScheduler
    - org.workflowsim.scheduler.MaxMinScheduler
    - org.workflowsim.scheduler.MCTScheduler
    - org.workflowsim.scheduler.MinMinScheduler
    - org.workflowsim.scheduler.StaticScheduler
  - org.workflowsim.clustering.BasicClustering (implements org.workflowsim.clustering.ClusteringInterface)
    - org.workflowsim.clustering.balancing.BalancedClustering
    - org.workflowsim.clustering.BlockClustering
    - org.workflowsim.clustering.HorizontalClustering
    - org.workflowsim.clustering.VerticalClustering
  - org.cloudbus.cloudsim.provisioners.BwProvisioner
    - org.cloudbus.cloudsim.provisioners.BwProvisionerSimple
  - org.cloudbus.cloudsim.Cloudlet
    - org.cloudbus.cloudsim.network.datacenter.NetworkCloudlet (implements java.lang.Comparable<T>)
    - org.workflowsim.Task
      - org.workflowsim.Job
  - org.cloudbus.cloudsim.lists.CloudletList
  - org.cloudbus.cloudsim.CloudletScheduler
    - org.cloudbus.cloudsim.CloudletSchedulerSpaceShared
    - org.cloudbus.cloudsim.CloudletSchedulerTimeShared
      - org.cloudbus.cloudsim.CloudletSchedulerDynamicWorkload
    - org.cloudbus.cloudsim.network.datacenter.NetworkCloudletSpaceSharedScheduler
  - org.cloudbus.cloudsim.core.CloudSim
  - org.cloudbus.cloudsim.examples.CloudSimExample1
  - org.cloudbus.cloudsim.examples.CloudSimExample2
  - org.cloudbus.cloudsim.examples.CloudSimExample3
  - org.cloudbus.cloudsim.examples.CloudSimExample4
  - org.cloudbus.cloudsim.examples.CloudSimExample5
  - org.cloudbus.cloudsim.examples.CloudSimExample6
  - org.cloudbus.cloudsim.examples.CloudSimExample7
  - org.cloudbus.cloudsim.examples.CloudSimExample8
  - org.cloudbus.cloudsim.core.CloudSimTags
  - org.workflowsim.utils.ClusteringParameters
  - org.cloudbus.cloudsim.examples.power.Constants
  - org.cloudbus.cloudsim.Consts
  - org.cloudbus.cloudsim.DatacenterCharacteristics
  - org.cloudbus.cloudsim.DataCloudTags
  - org.cloudbus.cloudsim.core.DeferredQueue
  - org.cloudbus.cloudsim.network.DelayMatrix\_Float
  - org.workflowsim.clustering.balancing.metrics.DistanceVariance (implements org.workflowsim.clustering.balancing.metrics.BalancingMetric)
  - org.cloudbus.cloudsim.examples.power.planetlab.Dvfs
  - org.cloudbus.cloudsim.examples.power.random.Dvfs
  - org.cloudbus.cloudsim.util.ExecutionTimeMeasurer
  - org.cloudbus.cloudsim.distributions.ExponentialDistr (implements org.cloudbus.cloudsim.distributions.ContinuousDistribution)
  - org.workflowsim.failure.FailureGenerator
  - org.workflowsim.failure.FailureMonitor
  - org.workflowsim.failure.FailureRecord
  - org.cloudbus.cloudsim.File
  - org.cloudbus.cloudsim.FileAttribute
  - org.cloudbus.cloudsim.network.FloydWarshall\_Float
  - org.cloudbus.cloudsim.core.FutureQueue
  - org.cloudbus.cloudsim.distributions.GammaDistr (implements org.cloudbus.cloudsim.distributions.ContinuousDistribution)
  - org.cloudbus.cloudsim.network.GraphReaderBrite (implements org.cloudbus.cloudsim.network.GraphReaderIF)
  - org.cloudbus.cloudsim.HarddriveStorage (implements org.cloudbus.cloudsim.Storage)
    - org.workflowsim.ClusterStorage
    - org.cloudbus.cloudsim.SanStorage
  - org.cloudbus.cloudsim.examples.power.Helper
  - org.workflowsim.clustering.balancing.metrics.HorizontalRuntimeVariance (implements org.workflowsim.clustering.balancing.metrics.BalancingMetric)
  - org.cloudbus.cloudsim.Host
    - org.cloudbus.cloudsim.HostDynamicWorkload
      - org.cloudbus.cloudsim.power.PowerHost
        - org.cloudbus.cloudsim.power.PowerHostUtilizationHistory
    - org.cloudbus.cloudsim.network.datacenter.NetworkHost
  - org.cloudbus.cloudsim.lists.HostList
  - org.cloudbus.cloudsim.network.datacenter.HostPacket
  - org.cloudbus.cloudsim.HostStateHistoryEntry
  - org.workflowsim.clustering.balancing.metrics.ImpactFactorVariance (implements org.workflowsim.clustering.balancing.metrics.BalancingMetric)
  - org.cloudbus.cloudsim.InfoPacket (implements org.cloudbus.cloudsim.Packet)
  - org.cloudbus.cloudsim.examples.power.planetlab.IqrMc
  - org.cloudbus.cloudsim.examples.power.random.IqrMc
  - org.cloudbus.cloudsim.examples.power.planetlab.IqrMmt
  - org.cloudbus.cloudsim.examples.power.random.IqrMmt
  - org.cloudbus.cloudsim.examples.power.planetlab.IqrMu
  - org.cloudbus.cloudsim.examples.power.random.IqrMu
  - org.cloudbus.cloudsim.examples.power.planetlab.IqrRs
  - org.cloudbus.cloudsim.examples.power.random.IqrRs
  - org.cloudbus.cloudsim.Log
  - org.cloudbus.cloudsim.distributions.LognormalDistr (implements org.cloudbus.cloudsim.distributions.ContinuousDistribution)
  - org.cloudbus.cloudsim.examples.power.planetlab.LrMc
  - org.cloudbus.cloudsim.examples.power.random.LrMc
  - org.cloudbus.cloudsim.examples.power.planetlab.LrMmt
  - org.cloudbus.cloudsim.examples.power.random.LrMmt
  - org.cloudbus.cloudsim.examples.power.planetlab.LrMu
  - org.cloudbus.cloudsim.examples.power.random.LrMu
  - org.cloudbus.cloudsim.examples.power.planetlab.LrrMc
  - org.cloudbus.cloudsim.examples.power.random.LrrMc
  - org.cloudbus.cloudsim.examples.power.planetlab.LrrMmt
  - org.cloudbus.cloudsim.examples.power.random.LrrMmt
  - org.cloudbus.cloudsim.examples.power.planetlab.LrrMu
  - org.cloudbus.cloudsim.examples.power.random.LrrMu
  - org.cloudbus.cloudsim.examples.power.planetlab.LrrRs
  - org.cloudbus.cloudsim.examples.power.random.LrrRs
  - org.cloudbus.cloudsim.examples.power.planetlab.LrRs
  - org.cloudbus.cloudsim.examples.power.random.LrRs
  - org.cloudbus.cloudsim.examples.power.planetlab.MadMc
  - org.cloudbus.cloudsim.examples.power.random.MadMc
  - org.cloudbus.cloudsim.examples.power.planetlab.MadMmt
  - org.cloudbus.cloudsim.examples.power.random.MadMmt
  - org.cloudbus.cloudsim.examples.power.planetlab.MadMu
  - org.cloudbus.cloudsim.examples.power.random.MadMu
  - org.cloudbus.cloudsim.examples.power.planetlab.MadRs
  - org.cloudbus.cloudsim.examples.power.random.MadRs
  - org.cloudbus.cloudsim.util.MathUtil
  - org.cloudbus.cloudsim.network.datacenter.NetworkConstants
  - org.cloudbus.cloudsim.examples.network.NetworkExample1
  - org.cloudbus.cloudsim.examples.network.NetworkExample2
  - org.cloudbus.cloudsim.examples.network.NetworkExample3
  - org.cloudbus.cloudsim.examples.network.NetworkExample4
  - org.cloudbus.cloudsim.network.datacenter.NetworkPacket
  - org.cloudbus.cloudsim.NetworkTopology
  - org.cloudbus.cloudsim.examples.power.planetlab.NonPowerAware
  - org.cloudbus.cloudsim.examples.power.random.NonPowerAware
  - org.workflowsim.utils.OverheadParameters
  - org.workflowsim.utils.Parameters
  - org.cloudbus.cloudsim.distributions.ParetoDistr (implements org.cloudbus.cloudsim.distributions.ContinuousDistribution)
    - org.cloudbus.cloudsim.distributions.LomaxDistribution (implements org.cloudbus.cloudsim.distributions.ContinuousDistribution)
  - org.cloudbus.cloudsim.Pe
  - org.cloudbus.cloudsim.lists.PeList
  - org.cloudbus.cloudsim.provisioners.PeProvisioner
    - org.cloudbus.cloudsim.provisioners.PeProvisionerSimple
  - org.workflowsim.clustering.balancing.metrics.PipelineRuntimeVariance (implements org.workflowsim.clustering.balancing.metrics.BalancingMetric)
  - org.cloudbus.cloudsim.examples.power.planetlab.PlanetLabConstants
  - org.cloudbus.cloudsim.examples.power.planetlab.PlanetLabHelper
  - org.cloudbus.cloudsim.examples.power.planetlab.PlanetLabRunnerConsole
  - org.cloudbus.cloudsim.power.models.PowerModelCubic (implements org.cloudbus.cloudsim.power.models.PowerModel)
  - org.cloudbus.cloudsim.power.models.PowerModelLinear (implements org.cloudbus.cloudsim.power.models.PowerModel)
  - org.cloudbus.cloudsim.power.models.PowerModelSpecPower (implements org.cloudbus.cloudsim.power.models.PowerModel)
    - org.cloudbus.cloudsim.power.models.PowerModelSpecPowerHpProLiantMl110G3PentiumD930
    - org.cloudbus.cloudsim.power.models.PowerModelSpecPowerHpProLiantMl110G4Xeon3040
    - org.cloudbus.cloudsim.power.models.PowerModelSpecPowerHpProLiantMl110G5Xeon3075
    - org.cloudbus.cloudsim.power.models.PowerModelSpecPowerIbmX3250XeonX3470
    - org.cloudbus.cloudsim.power.models.PowerModelSpecPowerIbmX3250XeonX3480
    - org.cloudbus.cloudsim.power.models.PowerModelSpecPowerIbmX3550XeonX5670
    - org.cloudbus.cloudsim.power.models.PowerModelSpecPowerIbmX3550XeonX5675
  - org.cloudbus.cloudsim.power.models.PowerModelSqrt (implements org.cloudbus.cloudsim.power.models.PowerModel)
  - org.cloudbus.cloudsim.power.models.PowerModelSquare (implements org.cloudbus.cloudsim.power.models.PowerModel)
  - org.cloudbus.cloudsim.power.PowerVmSelectionPolicy
    - org.cloudbus.cloudsim.power.PowerVmSelectionPolicyMaximumCorrelation
    - org.cloudbus.cloudsim.power.PowerVmSelectionPolicyMinimumMigrationTime
    - org.cloudbus.cloudsim.power.PowerVmSelectionPolicyMinimumUtilization
    - org.cloudbus.cloudsim.power.PowerVmSelectionPolicyRandomSelection
  - org.cloudbus.cloudsim.core.predicates.Predicate
    - org.cloudbus.cloudsim.core.predicates.PredicateAny
    - org.cloudbus.cloudsim.core.predicates.PredicateFrom
    - org.cloudbus.cloudsim.core.predicates.PredicateNone
    - org.cloudbus.cloudsim.core.predicates.PredicateNotFrom
    - org.cloudbus.cloudsim.core.predicates.PredicateNotType
    - org.cloudbus.cloudsim.core.predicates.PredicateType
  - org.cloudbus.cloudsim.provisioners.RamProvisioner
    - org.cloudbus.cloudsim.provisioners.RamProvisionerSimple
  - org.cloudbus.cloudsim.examples.power.random.RandomConstants
  - org.cloudbus.cloudsim.examples.power.random.RandomHelper
  - org.workflowsim.reclustering.ReclusteringEngine
  - org.workflowsim.utils.ReplicaCatalog
  - org.cloudbus.cloudsim.ResCloudlet
  - org.cloudbus.cloudsim.lists.ResCloudletList
  - org.cloudbus.cloudsim.examples.power.RunnerAbstract
    - org.cloudbus.cloudsim.examples.power.planetlab.PlanetLabRunner
    - org.cloudbus.cloudsim.examples.power.random.RandomRunner
  - org.cloudbus.cloudsim.core.SimEntity (implements java.lang.Cloneable)
    - org.cloudbus.cloudsim.core.CloudInformationService
    - org.cloudbus.cloudsim.examples.CloudSimExample8.GlobalBroker
    - org.cloudbus.cloudsim.core.CloudSimShutdown
    - org.workflowsim.ClusteringEngine
    - org.cloudbus.cloudsim.Datacenter
      - org.workflowsim.DatacenterExtended
      - org.cloudbus.cloudsim.network.datacenter.NetworkDatacenter
      - org.cloudbus.cloudsim.power.PowerDatacenter
        - org.cloudbus.cloudsim.power.PowerDatacenterNonPowerAware
    - org.cloudbus.cloudsim.DatacenterBroker
      - org.cloudbus.cloudsim.power.PowerDatacenterBroker
      - org.workflowsim.WorkflowScheduler
    - org.cloudbus.cloudsim.network.datacenter.NetDatacenterBroker
    - org.cloudbus.cloudsim.network.datacenter.Switch
      - org.cloudbus.cloudsim.network.datacenter.AggregateSwitch
      - org.cloudbus.cloudsim.network.datacenter.EdgeSwitch
      - org.cloudbus.cloudsim.network.datacenter.RootSwitch
    - org.workflowsim.WorkflowEngine
    - org.workflowsim.WorkflowPlanner
  - org.cloudbus.cloudsim.core.SimEvent (implements java.lang.Cloneable, java.lang.Comparable<T>)
  - org.workflowsim.clustering.TaskSet
  - org.cloudbus.cloudsim.network.datacenter.TaskStage
  - org.cloudbus.cloudsim.examples.network.datacenter.TestExample
  - org.cloudbus.cloudsim.examples.power.planetlab.ThrMc
  - org.cloudbus.cloudsim.examples.power.random.ThrMc
  - org.cloudbus.cloudsim.examples.power.planetlab.ThrMmt
  - org.cloudbus.cloudsim.examples.power.random.ThrMmt
  - org.cloudbus.cloudsim.examples.power.planetlab.ThrMu
  - org.cloudbus.cloudsim.examples.power.random.ThrMu
  - java.lang.Throwable (implements java.io.Serializable)
    - java.lang.Exception
      - org.cloudbus.cloudsim.ParameterException
  - org.cloudbus.cloudsim.examples.power.planetlab.ThrRs
  - org.cloudbus.cloudsim.examples.power.random.ThrRs
  - org.cloudbus.cloudsim.network.TopologicalGraph
  - org.cloudbus.cloudsim.network.TopologicalLink
  - org.cloudbus.cloudsim.network.TopologicalNode
  - org.cloudbus.cloudsim.distributions.UniformDistr (implements org.cloudbus.cloudsim.distributions.ContinuousDistribution)
  - org.cloudbus.cloudsim.UtilizationModelFull (implements org.cloudbus.cloudsim.UtilizationModel)
  - org.cloudbus.cloudsim.UtilizationModelNull (implements org.cloudbus.cloudsim.UtilizationModel)
  - org.cloudbus.cloudsim.UtilizationModelPlanetLabInMemory (implements org.cloudbus.cloudsim.UtilizationModel)
  - org.cloudbus.cloudsim.UtilizationModelStochastic (implements org.cloudbus.cloudsim.UtilizationModel)
  - org.cloudbus.cloudsim.Vm
    - org.workflowsim.CondorVM
    - org.cloudbus.cloudsim.network.datacenter.NetworkVm (implements java.lang.Comparable<T>)
    - org.cloudbus.cloudsim.power.PowerVm
  - org.cloudbus.cloudsim.VmAllocationPolicy
    - org.cloudbus.cloudsim.network.datacenter.NetworkVmAllocationPolicy
    - org.cloudbus.cloudsim.power.PowerVmAllocationPolicyAbstract
      - org.cloudbus.cloudsim.power.PowerVmAllocationPolicyMigrationAbstract
        - org.cloudbus.cloudsim.power.PowerVmAllocationPolicyMigrationInterQuartileRange
        - org.cloudbus.cloudsim.power.PowerVmAllocationPolicyMigrationLocalRegression
          - org.cloudbus.cloudsim.power.PowerVmAllocationPolicyMigrationLocalRegressionRobust
        - org.cloudbus.cloudsim.power.PowerVmAllocationPolicyMigrationMedianAbsoluteDeviation
        - org.cloudbus.cloudsim.power.PowerVmAllocationPolicyMigrationStaticThreshold
      - org.cloudbus.cloudsim.power.PowerVmAllocationPolicySimple
    - org.cloudbus.cloudsim.VmAllocationPolicySimple
  - org.cloudbus.cloudsim.lists.VmList
    - org.cloudbus.cloudsim.power.lists.PowerVmList
  - org.cloudbus.cloudsim.VmScheduler
    - org.cloudbus.cloudsim.VmSchedulerSpaceShared
    - org.cloudbus.cloudsim.VmSchedulerTimeShared
      - org.cloudbus.cloudsim.VmSchedulerTimeSharedOverSubscription
  - org.cloudbus.cloudsim.VmStateHistoryEntry
  - org.cloudbus.cloudsim.distributions.WeibullDistr (implements org.cloudbus.cloudsim.distributions.ContinuousDistribution)
  - org.workflowsim.WorkflowParser
  - org.workflowsim.examples.WorkflowSimExample1
  - org.workflowsim.examples.WorkflowSimExample2
  - org.workflowsim.WorkflowSimTags
  - org.cloudbus.cloudsim.util.WorkloadFileReader (implements org.cloudbus.cloudsim.util.WorkloadModel)
  - org.cloudbus.cloudsim.distributions.ZipfDistr (implements org.cloudbus.cloudsim.distributions.ContinuousDistribution)

## Interface Hierarchy

- org.workflowsim.clustering.balancing.metrics.BalancingMetric
- org.workflowsim.clustering.ClusteringInterface
- org.cloudbus.cloudsim.distributions.ContinuousDistribution
- org.cloudbus.cloudsim.network.GraphReaderIF
- org.cloudbus.cloudsim.Packet
- org.workflowsim.planning.PlannerInterface
- org.cloudbus.cloudsim.power.models.PowerModel
- org.workflowsim.scheduler.SchedulerInterface
- org.cloudbus.cloudsim.Storage
- org.cloudbus.cloudsim.UtilizationModel
- org.cloudbus.cloudsim.util.WorkloadModel

## Enum Hierarchy

- java.lang.Object
  - java.lang.Enum<E> (implements java.lang.Comparable<T>, java.io.Serializable)
    - org.workflowsim.utils.ClusteringParameters.ClusteringMethod
    - org.workflowsim.utils.Parameters.FTCMethod
    - org.workflowsim.utils.Parameters.FTCMonitor
    - org.workflowsim.utils.Parameters.FTCFailure
    - org.workflowsim.utils.Parameters.SCHMethod
    - org.workflowsim.utils.Parameters.PLNMethod
    - org.workflowsim.utils.ReplicaCatalog.FileSystem

- Overview
- Package
- Class
- Use
- Tree
- Deprecated
- Index
- Help

- Prev
- Next

- Frames
- No Frames

- All Classes
